# The psychological costs of behavioral immunity following COVID-19 diagnosis

**DOI:** 10.1038/s41598-024-59408-6

**Published:** 2024-04-30

**Authors:** Derek P. Spangler, Evaline Y. Li, Gabriela S. Revi, Jennifer T. Kubota, Jasmin Cloutier, Nina Lauharatanahirun

**Affiliations:** 1 Department of Biobehavioral Health, Penn State University, University Park, Pennsylvania USA; 2 Department of Psychological and Brain Sciences, University of Delaware, Newark, Delaware USA; 3 Department of Political Science and International Relations, University of Delaware, Newark, Delaware USA; 4 Department of Biomedical Engineering, Penn State University, University Park, Pennsylvania USA

**Keywords:** Psychology, Human behaviour

## Abstract

Prior COVID-19 infection may elevate activity of the behavioral immune system—the psychological mechanisms that foster avoidance of infection cues—to protect the individual from contracting the infection in the future. Such “adaptive behavioral immunity” may come with psychological costs, such as exacerbating the global pandemic’s disruption of social and emotional processes (i.e., pandemic disruption). To investigate that idea, we tested a mediational pathway linking prior COVID infection and pandemic disruption through behavioral immunity markers, assessed with subjective emotional ratings. This was tested in a sample of 734 Mechanical Turk workers who completed study procedures online during the global pandemic (September 2021–January 2022). Behavioral immunity markers were estimated with an affective image rating paradigm. Here, participants reported experienced disgust/fear and appraisals of sickness/harm risk to images varying in emotional content. Participants self-reported on their previous COVID-19 diagnosis history and level of pandemic disruption. The findings support the proposed mediational pathway and suggest that a prior COVID-19 infection is associated with broadly elevated threat emotionality, even to neutral stimuli that do not typically elicit threat emotions. This elevated threat emotionality was in turn related to disrupted socioemotional functioning within the pandemic context. These findings inform the psychological mechanisms that might predispose COVID survivors to mental health difficulties.

## Introduction

COVID-19 infection incurs long-term, adverse effects on mental health and well-being^1–3^. These adverse effects included the harmful disruption of normal social and emotional activities during the global pandemic^4,5^. Experiencing a prior COVID infection appeared to make a bad situation worse, likely enhancing the interruptive effects of lockdowns and social distancing on daily social and emotional activities^6,7^. The mechanisms linking the coronavirus and such pandemic disruption could inform interventions targeting mental health difficulties in future outbreaks.

Prior COVID infection fosters adaptive biological immunity, in which antibodies are synthesized to promote a stronger future defense against the coronavirus^8^. Might COVID infection also foster *adaptive behavioral immunity*? The behavioral immune system refers to the psychological mechanisms, such as disgust, that promote avoidance of infection signals in the first place (e.g., person sneezing)^9–11^. By adaptive, we mean that the behavioral immune system could “learn” that infection threats are imminent or that the body is weak due to recent infection^12^. This may elevate tendencies to experience disgust as a protective strategy so that we do not get sick again. Adaptive behavioral immunity likely constitutes a form of associative disgust learning, involving evaluative conditioning mechanisms, that takes place around the time of infection^13^. Here, aversive COVID symptoms are paired with environmental cues, such that those cues by themselves are evaluated as more negative and disgusting, thus representing evaluative conditioning of a disgust appraisal. Associative disgust learning can also be overgeneralized toward safe stimuli such that learned disgust evaluations/responses are inappropriately elicited towards neutral stimuli that were not paired with the unconditional stimulus (e.g., COVID symptoms)^14^. Indeed, the overgeneralization of behavioral immunity responses toward neutral stimuli may be common in humans because it promotes a “better safe than sorry” disease avoidance strategy^15,16^. Taken together, prior COVID infection may promote adaptive behavioral immunity where, via associative learning mechanisms, disgust is amplified and overgeneralized across infection threat and neutral stimuli.

The notion of adaptive behavioral immunity following COVID has indirect support in a study examining illness generally but not COVID specifically^17^. In this study, recently ill participants attended more to and more readily avoided illness-signaling stimuli in cognitive-affective tasks. Only one study, to our knowledge, examined adaptive behavioral immunity following COVID infection^18^. These authors reported that individuals previously diagnosed with COVID rated themselves as being more vulnerable to infection on a self-report questionnaire. While this finding hints at up-regulated behavioral immunity, they did not specifically measure disgust, behavioral immunity’s core dimension. The prior study’s reliance on self-report, although practical, was unable to assess the precise emotional reactions underlying behavioral immunity. Participant ratings of standardized threat and neutral stimuli (e.g., images) in a controlled task could more reliably characterize the emotional “architecture” of post-COVID behavioral immunity^19,20^. For instance, such a paradigm could identify whether previously infected individuals overgeneralize disgust responses to neutral stimuli, or whether their disgust responses are only elicited toward infection threat stimuli. Up-regulated behavioral immunity might explain how COVID infection worsened *socioemotional function* during the global pandemic. Socioemotional function refers to the broad and often co-varying interpersonal and affective competencies/behaviors that support societal adjustment, well-being, and lowered risk for mental illness across the lifespan^21,22^. While the precise facets of good socioemotional function vary across studies, they generally include low internalizing symptoms (e.g., anxiety), good coping skills, and engagement in prosocial behaviors (e.g., regularly talking with friends) that synergistically influence one another^23–25^.The coronavirus pandemic negatively disrupted multiple facets of socioemotional function (e.g., loneliness, social isolation, stress, anxiety), leading to a potential increase psychopathology risk^26,27^—a phenomenon we refer to as *pandemic disruption.* We posit that contracting COVID-19 infection may have exacerbated the pandemic’s negative socioemotional impacts. Deleterious psychological effects of behavioral immunity are possible, given that heightened disgust has been related to anxiety disorders, negative emotional bias, and lower levels of health-promoting approach behaviors^28–34^. Overgeneralized disgust to neutral stimuli may be especially costly to socioemotional function during the pandemic because it is not context-appropriate or prototypical for threat-based emotions like disgust to be evoked by neutral stimuli. The tendency to negatively respond to safe stimuli as if they were threats, and the related overgeneralization of associative threat learning to neutral stimuli, are leading risk factors for impaired well-being and psychopathology^35–42^. Taken together, adaptive behavioral immunity, although potentially adaptive in preventing illness, may incur costs to socioemotional function during the pandemic.

To properly characterize adaptive behavioral immunity, it is critical to identify its precise emotional architecture, i.e., post-infection affective states and the underlying threat defense systems mediating those states. So far, we have grounded post-infection emotion (i.e., adaptive behavioral immunity) in the behavioral immune system, which is theorized to be a specialized threat defense system for avoiding infection threats that is separate^16^ from a system we label *harm avoidance.* The harm avoidance system mediates fear reactions, fear learning, and fight/flight responses to more immediate threats of physical injury such as predators and conspecifics with malicious intentions^19,43,44^. The actual separateness of the two systems is unclear since fear can be evoked to contamination concerns/stimuli and has also been related to disgust measures^45^. It is therefore possible that adaptive behavioral immunity is broad in the sense that it also recruits prototypical harm avoidance emotions like fear in order to foster disease-avoidance after infection.

Consistent with adaptive behavioral immunity that is psychologically costly, we predicted that elevated behavioral immunity markers will mediate the link between prior COVID diagnosis and pandemic disruption (H1). Previously diagnosed individuals, relative to their never-diagnosed counterparts, will report higher disgust/sickness appraisals to infection threat and neutral stimuli, and those elevated markers will be related to greater pandemic disruption. Consistent with overgeneralized threat responses posing psychological risk, behavioral immunity responses to neutral relative to infection threat stimuli will have a stronger mediating effect in the pathway (H2). The specificity of adaptive behavioral immunity was tested with two competing hypotheses. Prototypical harm avoidance markers—fear/harm appraisals—could either mediate (H3a) or not mediate (H3b) the association with prior COVID diagnosis and pandemic disruption, which would support a broad (H3a) versus specific (H3b) recruitment of threat emotions in adaptive behavioral immunity.

Hypotheses were tested in a large sample of Mechanical Turk (MTurk) workers using an affective image rating task. Here, behavioral immunity markers were estimated with disgust ratings and sickness appraisals towards images depicting: (1) threat of infection, (3) threat of immediate harm, and (3) neutral stimuli. Harm avoidance markers were assessed as fear ratings and harm appraisals toward the same images. Participants self-reported everyday socioemotional disruption and prior COVID diagnosis via questionnaires. Hypotheses were not pre-registered. Our study may therefore be considered exploratory in nature, although hypotheses were generated a priori.

## Results

**Figure 1:**
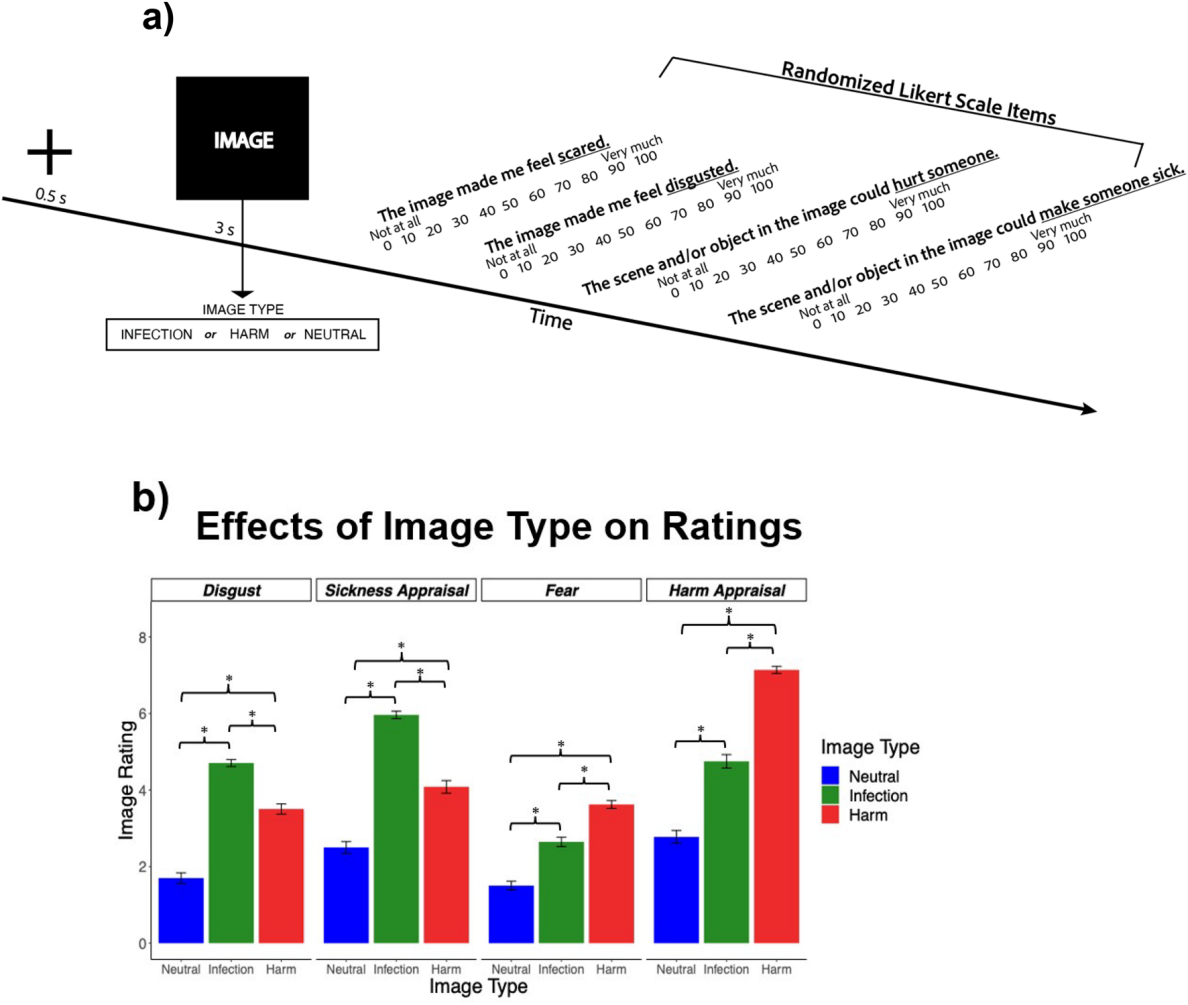
(**a**) Affective image rating task: A typical trial is presented. (**b**) Effects of image type on ratings: Means and 95% CIs are presented in the bar plot. *Statistically significant (two-tailed, *p* < 0.05) differences in ratings between image types. Differences between image types were tested in multilevel models as fixed effects (*β*) of dummy code contrasts on rating measures. See Supplemental Materials for the detailed method and results.

MTurk workers first self-reported their prior COVID diagnosis history and then their level of pandemic disruption via online questionnaires. Table 1 contains descriptive statistics for demographic factors and questionnaire measures. Participants then completed the affective image task (Fig. 1a). In this task, each participant rated 90 different images (30 threat of infection, 30 threat of harm, 30 neutral) on four Likert statements capturing prototypical behavioral immunity and harm avoidance markers. See Fig. 1b for mean rating differences between the image type conditions. Multilevel models tested differences in mean ratings and thus examined the effectiveness of the image conditions in activating the behavioral immune and harm avoidance systems (see Supplemental Materials for details on the method and results). All four negative ratings were higher for threat images compared to the neutral images, suggesting that the image paradigm was effective at broadly eliciting threat-based emotions (see Neutral vs. Infection and Neutral vs. Harm contrasts in Additional file 2: Table S2). Not surprisingly, the infection cue images more effectively activated behavioral immunity compared to the harm cue images, as disgust and sickness appraisals were significantly higher following the infection versus harm cue images (see Infection vs. Harm contrasts in Additional file 2: Table S2). The harm cue images were more effective at activating the harm avoidance system, since fear and harm appraisals were significantly elevated to harm versus infection images (see Infection vs. Harm contrasts in Additional file 2: Table S2). Those findings support the effectiveness and separateness of the image type conditions in activating the behavioral immune system versus the harm avoidance system.

**Table 1:** Descriptive statistics.

Variable	*N*	Mean (%)	SD	Minimum	Median	Maximum
*Age*	–	39.57	11.12	18	37	78
*Gender*	734					
Male	413	56.27%				
Female	320	43.6%				
Non-binary	1	0.14%				
*Race*						
African American/Black	74	10.08%				
Asian	31	4.22%				
Caucasian/White	612	83.38%				
Native American/Pacific Islander	7	0.95%				
Biracial/Multiracial/Other	10	1.36%				
*Ethnicity*						
Non-Hispanic	652	88.83%				
Hispanic	82	11.17%				
*Income*						
Less than $5,000	27	3.68%				
$5,000 through $11,999	50	6.81%				
$12,000 through $15,999	30	4.09%				
$16,000 through $24,999	74	10.08%				
$25,000 through $34,999	114	15.53%				
$35,000 through $49,999	140	19.07%				
$50,000 through $74,999	182	24.8%				
$75,000 through $99,999	89	12.13%				
$100,000 and greater	28	3.81%				
*Educational Attainment*						
Less than high school	2	0.27%				
High school diploma or equivalency	135	18.39%				
Associate’s degree or junior college	86	11.72%				
Bachelor’s degree or 4-year trade school	385	52.45%				
Postgraduate degree (e.g., PhD, MD)	126	17.17%				
*COVID Diagnosis History*						
Never diagnosed	597	81.34%				
Previously diagnosed	137	18.66%				
*Pandemic Disruption*	734	3.48	0.87	1	3.67	5

### H1. Elevated behavioral immunity mediates the association between prior COVID diagnosis and greater pandemic disruption

**Figure 2:**
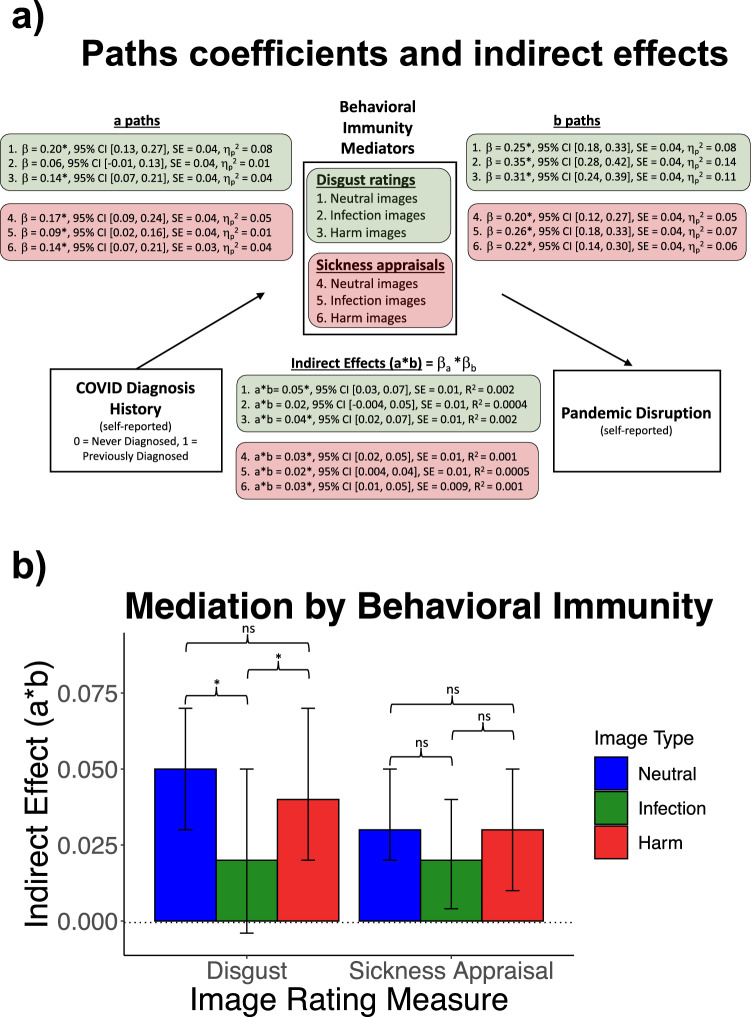
(**a**) Path coefficients and indirect effects for prototypical behavioral immunity ratings (disgust, sickness appraisals). (**b**) Indirect effects (a * b) of behavioral immunity ratings by image type. Whiskers indicate two-tailed 95% CIs from a wild bootstrap (10,000 iterations). *Statistically significant (two-tailed, *p* < 0.05) differences in indirect effects between image types. Differences were tested with a wild bootstrap (10,000 iterations). ns denotes non-significant differences (*p* > 0.05) in the indirect effects.

Indirect (i.e., mediational) effects were tested using the product (*a* * *b*) method^46^. Specifically, *a* paths (*β*_*a*_*)* tested the effects of COVID diagnosis history (dummy variable, 0 = never diagnosed, 1 = previously diagnosed) on image ratings, which tested mean differences in ratings between diagnosis history groups. The *b* paths (*β*_*b*_*)* tested the effects of image ratings on pandemic disruption. The individual paths and the products of *a* and b paths (*β*_a_*β_*b*_) were tested against zero with a wild bootstrap approach (10,000 iterations). The bootstrapped distributions generated two-tailed 95% CIs, and effects were deemed statistically significant (*p* < 0.05) if the CIs excluded zero. See Fig. 2 for path coefficients, indirect effects, and 95% CIs. Except for disgust ratings to infection images (which did not exhibit a significant mediational effect), *indirect effect* (*β*_*a*_ * *β*_*b*_) = 0.02, *p* > 0.05, *R*^2^ = 0.0004, disgust ratings and sickness appraisals to each image type mediated the association between COVID diagnosis history and pandemic disruption, *indirect effects* (*β*_*a*_ * *β*_*b*_) = 0.02 to 0.05, *p*s < 0.05, *R*^2^ = 0.0005 to 0.002. Except for the *a* path estimating the link between COVID diagnosis and disgust to infection images, *β* = 0.06, *p* > 0.05, η_p_^2^ = 0.01, all *a* paths (COVID → disgust and sickness appraisal ratings) were statistically significant, *β*s = 0.09 to 0.20, *p*s < 0.05, η_p_^2^ = 0.01 to 0.08. In addition, all *b* paths (disgust and sickness appraisal ratings → pandemic disruption) were statistically significant, *β*s = 0.20 to 0.35, *p*s < 0.05, η_p_^2^ = 0.05 to 0.14. Overall, these results mostly confirm the hypothesis (H1) that prototypical behavioral immunity markers mediate the association between COVID diagnosis and pandemic disruption. Relative to those never diagnosed, individuals previously diagnosed with COVID generally reported higher disgust and higher appraisals of sickness across the image types. The higher ratings among previously diagnosed individuals, regardless of image type, were in turn related to greater pandemic disruption. The exceptions to this pattern were the findings in which: (1) COVID diagnosis history was surprisingly unrelated to disgust to infection images (2) these ratings did not mediate the diagnosis-disruption association. Despite being non-significant, the size of the mediational effect for disgust to infection images (*R*^2^ = 0.0004) was similar to the mediational effect size for sickness appraisals to infection images (*R*^2^ = 0.0005).

### H2. Behavioral immunity activation to neutral relative to infection images more strongly mediates the association between prior diagnosis and greater pandemic disruption

Differences in mediation between image types were tested by bootstrapping (10,000 iterations, wild bootstrap) the raw differences between the indirect effect products (*β*_*a*_ * *β*_*b*_) in H1. The bootstrapped distributions generated two-tailed 95% CIs, which were used for significance testing the differences against zero. As hypothesized, disgust ratings to neutral images had a stronger mediating effect than disgust ratings to infection threat images, *difference* = 0.03, *95% CI* [0.001, 0.06], *R*^2^_*neutral*_ − *R*^2^_*infection*_ = 0.0016 (Fig. 2b). Contrary to our prediction, the mediating effect of sickness appraisals to neutral images was not statistically different from the mediating effect of sickness appraisals to infection images, *difference* = 0.01, *95% CI* [− 0.01, 0.03], *R*^2^_*neutral*_ – *R*^*2*^_*infection*_ = 0.0005. Additional file 2: Table S1 contains the differences in the indirect effects and path coefficients between image types.

### H3. Prototypical harm avoidance markers mediate the association between prior COVID diagnosis and greater pandemic disruption

**Figure 3:**
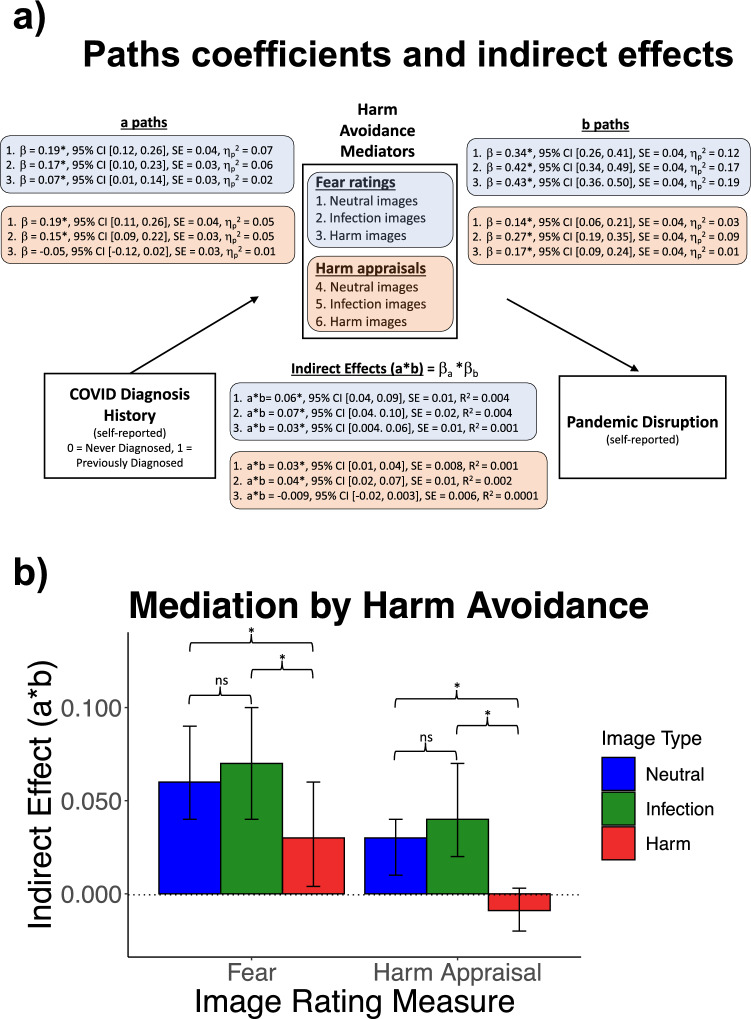
(**a**) Path coefficients and indirect effects for prototypical harm avoidance ratings (fear, harm appraisals). (**b**) Indirect effects (a * b) of harm avoidance ratings by image type. Whiskers indicate two-tailed 95% CIs from a wild bootstrap (10,000 iterations). *Statistically significant (two-tailed, *p* < 0.05) differences in indirect effects between image types. Differences were tested with a wild bootstrap (10,000 iterations). ns denotes non-significant differences (*p* > 0.05) in the indirect effects

The same statistical approach for H1 was employed to test mediation by the harm avoidance ratings. Figure 3 contains the path coefficients, indirect effects, and two-tailed 95% CIs. Fear ratings to each image type significantly mediated the relationship between COVID diagnosis history and pandemic disruption, *indirect effects* = 0.03 to 0.07, *p*s < 0.05, *R*^2^ = 0.001 to 0.004. Harm appraisals to neutral and infection images, *indirect effects* = 0.03 to 0.04, *p*s < 0.05, *R*^2^ = 0.001 to 0.002, but not harm appraisals to harm images, *indirect effect* = -0.009, *p* > 0.05, *R*^2^ = 0.0001, significantly mediated the association between diagnosis history and pandemic disruption. Within the significant mediational effects for fear and harm appraisals, the* a* paths, *β*s = 0.07 to 0.19, *p*s < 0.05, η_p_^2^ = 0.02 to 0.07, and *b* paths, *β*s = 0.14 to 0.43, *p*s < 0.05, η_p_^2^ = 0.03 to 0.19, were statistically significant. The findings support the hypothesis (H3a) that prototypical harm avoidance markers also mediate the link between COVID diagnosis and pandemic disruption. Specifically, relative to those never diagnosed, individuals previously diagnosed with COVID reported higher fear and higher harm appraisals across all image types— except for harm ratings to images depicting harm cues. These higher ratings among previously diagnosed individuals were in turn related to greater pandemic disruption. The results suggest that elevated behavioral immunity and its “psychological costs” following COVID diagnosis may involve broad activation of threat responses as opposed to narrow activation of disgust and disgust-related responses (e.g., sickness appraisals).

## Discussion

Prior research has garnered support for “adaptive behavioral immunity” where a prior infection elevates psychological defenses such as disgust to prevent future illness^17^. This mechanism, although beneficial for physical health, may be costly to socioemotional functioning, as the heightened expression of threat responses (e.g., disgust) is a risk factor for psychopathology and impaired well-being^33,47^. The existence of adaptive behavioral immunity after COVID-19 could inform understanding of mental health risk in its survivors. The current study used an image rating task to investigate this adaptive yet costly mechanism as a mediational pathway: COVID diagnosis → emotional image ratings → disruption. Our results for this mediational pathway are consistent with a costly form of adaptive behavioral immunity following COVID-19 infection. Specifically, individuals with a history of COVID diagnosis, relative to the those with no such history, generally have (1) stronger behavioral immune system responses across varied stimuli, including neutral stimuli that should not elicit threat responses, and (2) in turn exaggerated interruptions to daily socioemotional life by the pandemic context (e.g., lockdowns, closures, isolation). This elevated behavioral immunity appears to broadly recruit threat responses prototypically linked to the behavioral immunity (e.g., disgust) and harm avoidance (e.g., fear) systems. It must be noted that our results are cross-sectional and correlational. Therefore, the causal role of coronavirus infection and the image ratings must be cautiously interpreted. Our study still provides novel evidence that an earlier infection is related to both exaggerated threat responses and daily psychological functioning in the same pathway. This sheds light on potential emotional and cognitive abnormalities in COVID survivors that could place them at increased risk for various psychological and social difficulties.

The current findings meaningfully add to a small body of existing research that has documented a phenomenon we label “adaptive behavioral immunity.” Miller and Maner (2012) reported that participants with any recent illness more strongly attended to and avoided illness-signaling images, with effect sizes of η_p_^2^ = 0.11 and 0.15. In a more COVID-relevant study^18^, individuals who had been previously diagnosed with COVID reported that they felt more vulnerable to illness, with an effect size of η_p_^2^ = 0.005. In the current study, we found that the associations between prior COVID diagnosis and threat ratings to the images (*a* paths) were η_p_^2^ = 0.01 to 0.08, which are slightly smaller than Miller and Maner (2012) but notably larger than that of Troisi et al., (2023). The ranges of the effect sizes could be classified as small to medium based on Cohen’s (1988) rule of thumb, with our mediational effects being interpreted as small because our *R*^2^ values were below 0.01^48^.The relations between threat ratings and pandemic disruption (*b* paths) had a larger range such that effects could be classified small to large^48^. Our range of a paths (COVID → ratings) being larger than Troisi et al. may be due to our reliance on a controlled task measure of behavioral immunity as opposed to a self-reported questionnaire. The generally small size of many of our effects suggests that up-regulated behavioral immunity following COVID infection might be overshadowed by the more obvious physical issues that are more closely related to “long COVID syndrome”^4^. Despite their small size, the observed effects may still have incremental and practical impacts on the quality of life of COVID survivors who must cope with a mélange of social, physical, and emotional difficulties in their daily lives^1^.

### Potential neurophysiological foundations

Numerous manifestations of biological immunity can be observed through perception and behavior^49^, with many of these interactions being reliant on functionally interconnected neural structures including the amygdala, insular cortex, and ventromedial nucleus of the hypothalamus^50,51^. Speaking to a connection between biological and behavioral immunity, those regions are also implicated in the coordination, generation, and learning of threat responses including disgust and fear^52,53^. The insular cortex might play an especially important role in behavioral-biological immunity interaction, as it contributes to the encoding/retrieving of specialized immunological responses^54^, affective evaluations that support negative emotional biases^55,56^, and the integration of (afferent) bodily and (efferent) brain signals^57^. We speculate that our current pattern of increased threat activation among COVID survivors relies on these neurophysiological structures, especially the insular cortex, such that they provide a substrate for adaptive behavioral immunity (a form of biological-behavioral immunity interaction). For example, prior immunological responses related to earlier COVID infection might be encoded in insular cortex neurons, which prime the activation of neuronal populations across a distributed “threat” network that includes the insula, hypothalamus, and amygdala^52^. This may in turn lead to biased and indiscriminate disgust/fear responding across varied environmental stimuli, even neutral ones (further discussed below).

The priming of behavioral immunity in the insula and other neural regions likely reflects associative disgust/fear learning that has been documented in the prior literature^13,58^. Here, the firsthand negative experiences and symptoms associated with infection elicit threat evaluations and heightened stress^59^. These symptoms/experiences are feasibly paired with environmental cues that signal illness (e.g., person coughing) such that those environmental cues by themselves evoke even stronger negative affective evaluations (e.g., heightened disgust) than they did before the illness. Indeed, consolidation and storage of threat learning relies on insular neurons^60^. More research is needed to study the specific neural interactions that underlie communication between biological immunity, behavioral immunity, and threat learning, and future research is needed to substantiate the proposed neurophysiological explanations.

### Importance of overgeneralization

The overgeneralization of behavioral immunity to threat-absent neutral stimuli represents a “better safe than sorry” bias that is heightened in situations that threaten health and survival^61^. This functional account may explain our finding where COVID diagnosis was related to heightened behavioral immunity markers toward neutral images. Here, a recent coronavirus infection may enhance the overgeneralization of threat responses towards safety to increase prophylactic efficacy, widen spatiotemporal distance from infection threats, and increase survival odds^9,16^. Heightened neutral-stimulus reactions may also pose costs to socioemotional functioning; exaggerated threat responses to safety are inherently context-inappropriate and are key etiological factors in psychopathology^35,38^. This idea stimulated our hypothesis that behavioral immunity to neutral versus infection stimuli would more strongly mediate the diagnosis-disruption relationship. Although we found support for this hypothesis, follow-up contrasts of the b paths (Supplemental Materials) do not support neutral ratings being more strongly related to pandemic disruption. The stronger mediation of neutral ratings was instead driven by COVID history having a stronger relation to neutral ratings than it did to infection ratings (i.e., *a* paths). As a potential explanation, behavioral immunity following COVID might notably activate disgust to neutral stimuli because neutral reactivity provides maximal changes in affective biases and later motivational actions that enhance survival. Supporting this interpretation, neutral stimuli are believed to be more emotionally ambiguous and thus more subject to emotional biases than “strong” stimuli such as obvious threats^62,63^. This idea is consistent with our findings that prior COVID diagnosis was least related to threat ratings to infection and harm images, the images that should most strongly evoke threat responses. Taken together, neutral stimuli may operate as a “blank slate” that allow emotional biases to emerge more easily. Like our interpretation above, this overgeneralization likely implicates an associative learning mechanism whereby threat evaluations to the unconditioned stimulus (COVID symptoms/experiences) are initially paired with infection signals such as the sight of sneezing or coughing. It is likely that such conditioned disgust/fear evaluations, like other threat responses that are learned, are inappropriately overgeneralized to neutral stimuli that do not signal infection risk—such that neutral stimuli by themselves elicit threat evaluations^14,42^.

### (Adaptive) behavioral immunity is more than disgust

Our findings challenge behavioral immunity as a narrow phenomenon merely limited to disgust and disgust-related cognitions about illness and contamination^9,10,16,64^. They suggest that prior illness recruits a broader repertoire of defense-based emotions like fear to putatively support pathogen avoidance, as has been speculated by others^65^. Our findings are not surprising given that avoiding infection may represent a type of harm avoidance. Pathogens like coronavirus can indeed elicit physical harm such as fever, organ damage, and even death^66^. Yet, it could be argued that infection cues pose physical harm that is less immediate (it takes time for the pathogen to have an effect) and less obvious (pathogens are often microscopic) than predators^16^. Additional research is needed to examine physiological, neural, motoric, and psychological features that may or may not distinguish these defensive threat systems.

### Limitations, future directions, and conclusions

As noted above, the results are cross-sectional and not necessarily indicative of adaptive behavioral immunity or of prior infection having causal effects on emotion. A related limitation is that self-reported diagnosis was used, despite the possibility that some individuals were unaware that they were COVID-positive. It is also unclear how participants arrived at their diagnosis, which is important information given that diagnostic tests exhibit notable variability in their reliability/validity. Future longitudinal studies should corroborate our results using antibody diagnostic tests administered in the clinic. Relatedly, the current study did not measure COVID-19 infection or symptoms in terms of their precise timing or severity. We were thus unable to disentangle prior versus current infection status in the current study, preventing us from testing whether currently infected individuals had stronger threat responses and pandemic disruption. This is a likely possibility given that feeling sick can involve increased negative emotion^59^. Our analyses were also unable to separate individuals with high versus low severity symptoms who might encounter heightened threat emotions and socioemotional difficulties. Indeed, there is immense inter-person variability in COVID symptom severity that remains poorly understood^67^. In particular, the cross-sectional mediation analyses used in this study should be replicated in future studies with longitudinal designs to help us better understand the causal relationships among COVID infection, threat responses, and behavioral immunity. Furthermore, disgust and fear activate neurophysiologic and motoric response systems that do not always align with subjective emotion reports^68,69^. Future work therefore needs to examine the effects of COVID diagnosis on neural and psychophysiological measures in controlled laboratory settings. Lastly, some of our interpretations involved associative learning processes, which we did not directly model here given that we did not use a learning paradigm. Future research on adaptive behavioral immunity might examine COVID infection’s impacts on direct measures of associative learning where, for example, a conditioned visual stimulus is paired with an unconditioned stimulus that reflexively activates biological and/or behavioral immunity reactions. This study would directly test whether prior infection strengthens the conditioned associations between emotional and immunological responses to environmental cues. Such research could elucidate the precise mechanisms of adaptive behavioral immunity.

Despite the caveats and need for future research, the current study is the first to show that a positive COVID diagnosis is associated with elevated threat responses and disrupted socioemotional functioning. The current research may expand the definition of “long COVID syndrome” beyond mere physical/somatic symptoms so that it also includes psychological alterations, i.e., elevated defensive reactivity to the environment. Such elevations may be cumbersome to the ebb and flow of daily social and emotional experiences. Our findings also have implications for future health prevention/intervention efforts. It is estimated that a similar global pandemic is likely to occur with a 38% probability in one’s lifetime^70^. As suggested by the current findings, elevated threat responses may be a promising target for predicting and treating mental health difficulties that occur in response to recent infections in future pandemics.

## Methods

### Participants

Participants were adult workers (at least 18 years old) on the Amazon Mechanical Turk (MTurk) platform, an online system used to crowdsource human responses, including those for scientific research. Participants were required to be English-fluent residents of the United States with no history of mental illness. These criteria were assessed with an online screening questionnaire. Nine hundred and twelve participants were enrolled in the study. Eighty-six individuals were excluded because they failed at least one attention check question that was embedded in the image rating task. Fifty-five additional participants were excluded due to substantially low variance in the image ratings (*SD* < 10), a pattern suggestive of bot responses or careless responding. Twelve participants were excluded because their survey responses could not be aligned with their image responses (either due to the participant’s failure to provide a survey code at the end of the study or erroneous duplication of subject identifiers). Of the remaining 759 participants, 25 individuals were excluded because they failed to complete the online demographic questionnaires. The remaining 734 participants served as the final sample that was included in all analyses. Table 1 contains the demographic characteristics of the final sample. All study procedures were approved by and are in accord with the Institutional Review Board (IRB) at Penn State University. Informed consent was attained from all participants before study procedures.

### Procedure

The study took place during the global COVID-19 pandemic between September 15, 2021 and January 24, 2022. The study coincided with the appearance of the Omicron variant (December 2021) and the peak number of cases in the United States (~ January 14, 2022)^71^. All components of this online study were conducted remotely via participants' personal electronic devices. The screening survey, all study questionnaires, and the image task were implemented on Qualtrics XM software^72^. Participant response data were stored on the Qualtrics password-protected online cloud server and were later downloaded for offline analysis.

Prior to being enrolled in the study, MTurk workers saw the study as a Human Intelligence Task (HIT) on Amazon MTurk and accepted it by clicking on the weblink. Workers were then provided with an online consent form and screening questionnaire. If informed consent was obtained and the participant passed all screening criteria, then Workers were officially enrolled in the study.

Participants were next provided with a weblink that, once clicked, progressed them through all study components. They first completed a brief demographics and health history survey followed by the pandemic disruption survey. Participants next completed the affective image rating task, which lasted approximately one hour. After the image task, participants were provided with a random 5-digit survey code to input into MTurk and receive compensation of $6, delivered electronically by Amazon.

### Affective image rating task

The task displayed images depicting scenes aimed to elicit subjective emotional responses. The images were selected and downloaded/purchased from Shutterstock, Pexels, Dreamstime, and Wikimedia Commons, which are online image providers of professional, royalty-free and/or public domain stock photography. All images were standardized by resizing them to a 1:1 aspect ratio and a resolution of 500 × 500 pixels (see Supplemental Materials for image stimuli).

The task was composed of three repeated-measures image type conditions: (1) Neutral (e.g., landscapes, everyday objects), (2) Threat of infection (e.g., blood, mucus, feces), (3) Threat of bodily harm (e.g., weapons, dangerous animals, interpersonal violence). The image conditions/stimuli were defined a priori based on their pre-existing stimulus content as opposed to the emotions they are hypothesized to elicit a posteriori. This is why we do not refer to infection threat images as “disgust-eliciting stimuli,” for example, although infection threat cues were predicted to more strongly elicit disgust relative to threat-of-harm and neutral cues. Nevertheless, there may be concern that each harm image set contains five images depicting venomous creatures (i.e., snakes or spiders), which have been shown to elicit subjective disgust. We therefore excluded the snake/spider images and reran the harm image models in supplemental analyses (Supplemental Materials). All of harm image results had similar effect sizes and the same statistical conclusions as what is reported in the main text. The primary results in the main text include all images.

Each image was rated by the participant on the disgust and fear levels they felt while viewing the image, and on the degree to which the image scene would cause them sickness and physical harm (sickness and harm appraisals). There were 30 trials for each image condition, yielding 90 trials in the task overall. The images were randomized across the 90 trials. Furthermore, participants were randomly assigned to one of three image sets, which were composed of 90 images each. The image sets generally conveyed the same affective content, in that they each had the same three image type conditions—neutral (N = 30), infection (N = 30), bodily harm (N = 30). They differed in the particular image scenes used such that none of the 90 images used in set 1 were used in set 2, for example. Image set was controlled for as between-subject variable in the analyses (see Statistical Analysis below). Three different sets were utilized to increase the heterogeneity and number of images in order to standardize the images for a future study.

A single trial of the task is detailed as follows (Fig. 1). A trial started with a 500-ms fixation cross. An image was next presented and remained on the screen for 3000 ms. After the image disappeared, participants were asked to rate four statements on Likert scales. The statements “The image made me feel disgusted.” and “The image made me feel scared.” were rated on scales ranging from 0 to 100, with 0 corresponding to “Not at all” and 100 corresponding to “Very much.” The statements “The scene and/or object in the image could make someone sick” and “The scene and/or object in the image could hurt someone” were rated on scales ranging from 0 to 100, with 0 corresponding to “Strongly disagree” and 100 corresponding to “Strongly agree.” All ratings were provided on a visual slider. Ratings were made by dragging the slider to an appropriate score on the scale, with lower ratings being closer to the left and higher ratings being closer to the right. The order of the four statements were randomized across trials. Once participants made their ratings, they proceeded to the next trial. For each participant, an attention check question was presented three times during the image rating task, such that the timing of these questions across trials was randomized. The attention check questions asked participants to move a slider to a single number (10, 40, or 70) on a continuous scale ranging from 0 to 100. Failure to move the slider to the correct number resulted in a failed attention check.

### Measures

#### Demographics

Participants self-reported demographic information via Qualtrics. Such demographic characteristics may confound associations among prior diagnosis, image ratings, and pandemic disruption. The following self-report measures were therefore included as covariates in the analyses below. Age was measured as a continuous variable in years. Gender was measured with a choice response to one of three “bubble” options (Male, Female, Other). If the participant chose “Other,” then they were asked to specify in a free response text box. Gender was represented as a binary dummy variable: 0 = Male or Other, 1 = Female. One participant self-reported an “Other” gender and was coded with males. Self-identifying as female is associated with increased threat responses to image stimuli^73^ and heightened risk for most psychopathologies^74^. For this reason, the female gender served as the reference group to which all other genders were compared, and the non-binary participant was also retained in the analysis to ensure maximal gender inclusivity^75^. Race was measured as choice response to one of six bubble options: (1) American Indian/Alaskan Native, (2) Asian, (3) Black/African-American, (4) Native Hawaiian/Other Pacific Islander, (5) White/Caucasian, (6) Other. To facilitate analysis, Race was represented as a binary dummy variable: 0 = White, 1 = Non-White. This coding was chosen given the many disproportionate health effects of COVID-19 on non-White Americans^76^. Hispanic ethnicity was measured with a “Yes” or “No” choice response, and was represented as a binary dummy variable (0 = No, 1 = Yes). Past-year income level was measured with a choice response to one of nine options: (1) Less than $5,000, (2) $5,000 through $11,999, (3) $12,000 through $15,999, (4) $16,000 through $24,999, (5) $25,000 through $34,999, (6) $35,000 through $49,999, (7) $50,000 through $74,999, (8) $75,00 to $99,999, and (9) $100,000 and greater. In the analysis, income level was represented as a continuous variable (0–8) in which higher scores represented relatively higher income. Lastly, education level was measured as one of the following: (1) Less than high school, (2) High school diploma or equivalency, (3) Associate’s degree or junior college, (4) Bachelor’s degree or four-year trade school, (5) Any degree beyond a Bachelor’s (Master’s, PhD, or medical/legal degree). Specifically, education was represented as a continuous variable (0–4) in which higher scores represented relatively higher education level.

#### COVID-19 diagnosis history

Diagnosis history was measured with the self-report questionnaire item: “Have you ever been diagnosed with COVID-19?” Participants provided a choice response of “Yes” or “No” by clicking the corresponding bubble. Diagnosis history was represented as a dummy binary variable: 0 = Never Diagnosed, 1 = Previously diagnosed.

#### Pandemic disruption

We developed a 10-item self-report questionnaire that aimed to measure the degree to which daily social and emotional functions were disrupted by the coronavirus pandemic. Participants rated the degree to which behaviors, thought patterns, and emotions were different during the pandemic relative to the time before the pandemic. These elements were represented as 10 different statements. The following statements estimated disruptions to emotional function. *Item 1*: “Since the COVID-19 pandemic, I have felt more anxious than usual.” *Item 2*: “Since the COVID-19 pandemic, I am more worried that I will get sick.” *Item 8*: “Since the COVID-19 pandemic, I have felt more stressed than usual.” *Item 10*: “Since the COVID-19 pandemic, I take less risks than usual.” These statements estimated disruptions to social behavior: Item 3: “Since the COVID-19 pandemic, I have been less social than usual.” Item 4: “Since the COVID-19 pandemic, I talk to strangers less.” Item 6: “Since the COVID-19 pandemic, I have been more likely to avoid physical contact with other people.” Item 7: “Since the COVID-19 pandemic, I have worked from home more often.” The following statements estimated emotional responses to social stimuli or lack thereof: Item 5: “Since the COVID-19 pandemic, I have felt more uneasy around people than usual.” Item 9: “Since the COVID-19 pandemic, I have felt more lonely than usual.” These three different functions are not meant to be orthogonal but instead reflective of interrelated processes that support one another and underlie risk for psychopathology^21,22^. Participants rated each statement on a 5-point Likert scale, with 1 corresponding to “Strongly disagree” and 5 corresponding to “Strongly agree.”

To ensure that the questionnaire reflects a unitary disruption construct, the scores were examined with an exploratory factor analysis using a minimum residual solution. A screen-test suggested a clear one factor solution in which the first factor had an *Eigenvariate* of 4.57 and the second “factor” had an *Eigenvariate* of 0.52. When fitting the single factor model, all items demonstrated logically consistent and moderately sized factor loadings, *r*s > 0.60, except Item 7 (“Since the COVID-19 pandemic, I have worked from home more often.”) which had a factor loading of *r* = 0.43. We therefore excluded this item; the factor analysis was conducted again and still yielded a clear one factor solution (*Eigenvariate* = 4.38) with logically consistent and high factor loadings, *r*s > 0.60. These results suggest a global disruption construct in the nine retained items. Those items were averaged to index *pandemic disruption* for each participant such that higher scores represented greater disruption.

#### Affective image ratings

Continuous (0–100) ratings to the statement “The image made me feel disgusted” were used to measure disgust levels. Analogous ratings to the statement “The scene and/or object in the image could make someone sick” indexed sickness appraisals.

Ratings to the statements “The image made me feel scared” and “The scene and/or object in the image could hurt someone” were used to measure fear levels and harm appraisals, respectively.

### Statistical analysis

#### Pre-analysis data processing

All variables exhibited approximately normal distributions except the image rating measures: disgust, sickness appraisals, fear, and harm appraisals. Deviations from normality are not a problem per se when using bootstrapping, which relaxes assumptions about the normality of model residuals (see below). However, the skew of the image ratings caused extreme outliers based on Tukey’s rule^77^—i.e., scores that were greater than (Quartile 3 + 3*interquartile range). The image rating variables were therefore submitted to Box-Cox transformations to normalize their distributions^78^. Optimal lambda values were estimated for each variable using a model fitting procedure with the MASS package in RStudio^79^. No other measures exhibited extreme outliers, i.e., values < (Quartile 1—3*interquartile range) or > (Quartile 3 + 3*interquartile range)^77^. All statistical analyses including those for mediation were performed on the Box-Cox transformed image ratings.

#### Manipulation check

Before testing hypotheses, we performed a manipulation check of the image task with multilevel regression models. The analysis tested, for example, whether the infection images had higher disgust ratings than neutral images. Disgust, sickness appraisals, fear, and harm appraisals were entered as dependent measures in their own models. A random intercept of participant was entered in each model to address the repeated-measures nature of the data. Two dummy codes were added as fixed effects to each model. The dummy codes tested the difference in ratings between neutral and infection images, and the difference in ratings between neutral and harm images. Models were fit in RStudio with restricted maximum likelihood using the *lme4* package^80^. Two-tailed 95% CIs were generated to statistically test the fixed effects. These results are depicted in Fig. 1B. A fuller description of the statistical analysis and the model results are provided in the Supplemental Materials.

### Hypothesis testing

The predicted indirect effects in H1 were tested with the product (a*b) method and bootstrapped confidence intervals^46,81^. This approach is more powerful that the traditional causal steps approach to mediation^82^ and can uncover meaningful indirect effects when the total effect (c path) is non-significant^83^.The indirect effect approach is especially useful here since the total effect of COVID diagnosis history on pandemic disruption was non-significant when adjusting for covariates (covariates detailed below), *β* = 0.06, *95% CI* [− 0.01, 0.13]. However, the total effect was statistically significant without covariates, *β* = 0.10, *95% CI* [0.04, 0.17]. Mediation can still occur and is theoretically important when the total effect is not statistically significant^83^.

Specifically, in testing H1, we first estimated the *a* (COVID diagnosis history → image ratings) and *b* (image ratings → pandemic) paths using separate multiple regression equations. The indirect effect—which tested the image ratings as a mediator in the association between diagnosis and disruption—was computed as the product of the* a* and *b* paths (*a* * *b*). To test the *a* * *b* product against zero, a sampling distribution in the statistic was generated with bootstrapping. Importantly, the COVID diagnosis history variable was unbalanced, such that 137 participants reported a previous COVID diagnosis, and 597 participants reported never having COVID. Therefore, a wild bootstrap (10,000 iterations) was used to derive the *a* * *b* sampling distribution, since the wild bootstrap addresses the unbalanced data and related heteroscedasticity in residuals^84^. From the bootstrapped distributions, two-tailed percentile 95% CIs were computed. An indirect effect (*a* * *b*) was deemed statistically significant if it did not include zero in the CI. Paths and indirect effects were estimated separately for each combination of rating measure (disgust, sickness appraisals) and image type (neutral, infection, harm), yielding 6 different mediational tests. The separate approach was necessary to test the overgeneralization part of the hypothesis, i.e., that behavioral immunity markers would be higher in response to *each* image type. In each regression model that tested *a* and *b* paths, demographic variables of gender, age, race, ethnicity, income, education were entered as covariates. The image set condition (1–3) was represented with two dummy code variables where the first image set served as the reference group. Adjusting for those covariates ensured that the indirect effects and their paths were not confounded by pre-existing attributes or by the image set to which participants were assigned. These analyses were conducted in RStudio using the *lmboot* package^85^ and base R functions.

For H2, we sought to test whether disgust/sickness to neutral images had a stronger mediating effect than the same ratings to infection images. To test H2, we statistically compared the size of the indirect effects (*a* * *b*) that were estimated for H1. The differences in *a* * *b* values between neutral and infection images was computed and submitted to a wild bootstrap (10,000 iterations) to generate two-tailed 95% CI. If zero was not included in the CI, then there was a statistically significant difference in the size of the mediational effect between neutral and infection images. H3 was concerned with the mediational effects of prototypical harm avoidance markers. H3 was tested with the same approach that was used to test H1, except harm avoidance metrics (fear and sickness appraisal ratings) served as mediators in the models.

Effect size magnitudes were computed for all paths and indirect effects. Specifically, partial eta squared were computed for each a and b path, and *R*^2^ was computed for each indirect effect. *R*^2^ values were calculated as the product of the squared partial correlations for the a and b paths (a * b) (Preacher & Kelley, 2011). Lastly, effect sizes for the difference in mediation between image types were calculated as the differences in the *R*^2^ values that were described above, e.g., *R*^2^_*neutral*_ −* R*^2^_*infection.*_

### Supplementary Information

Supplementary Information 1.

Supplementary Information 2.

## Data Availability

The datasets and code used to perform the statistical analyses can be accessed in the supplementary information files.
