# Supplementary material for: The psychological costs of behavioral immunity following COVID-19 diagnosis
Source: Sci Rep. 2024 Apr 30;14:9899. doi: 10.1038/s41598-024-59408-6 (PMC11061184; doi:10.1038/s41598-024-59408-6)
Supplement: Supplementary file 1 — Supplementary Information 1. [file 41598_2024_59408_MOESM1_ESM.docx]

Pandemic Disruption Questionnaire

1. Since the COVID-19 pandemic, I have felt more anxious than usual.
2. Since the COVID-19 pandemic, I have been less social than usual.
3. Since the COVID-19 pandemic, I talk to strangers less.
4. Since the COVID-19 pandemic, I have felt more uneasy around people than usual.
5. Since the COVID-19 pandemic, I have been more likely to avoid physical contact with other people.
6. Since the COVID-19 pandemic, I have worked from home more often.
7. Since the COVID-19 pandemic, I have felt more stressed than usual.
8. Since the COVID-19 pandemic, I have felt more lonely than usual.
9. Since the COVID-19 pandemic, I take less risks than usual.
10. Since the COVID-19 pandemic, I am more worried that I will get sick.
