# Supplementary material for: The psychological costs of behavioral immunity following COVID-19 diagnosis
Source: Sci Rep. 2024 Apr 30;14:9899. doi: 10.1038/s41598-024-59408-6 (PMC11061184; doi:10.1038/s41598-024-59408-6)
Supplement: Supplementary file 2 — Supplementary Information 2. [file 41598_2024_59408_MOESM2_ESM.docx]

**Supplemental Materials**

**Ratings Differ between the Image Type Conditions**

Supplemental Table 1 contains descriptive statistics for the rating measures as a function of image type: neutral, infection, harm. The effects of the image task were represented as differences in mean ratings between the image conditions. These differences were tested with two-level multilevel regression models that were fit to the trial-level data. The Box-Cox transformed rating measures served as dependent measures in the models, as the transformations helped normalize their distributions. Separate models were conducted for each dependent measure: disgust, sickness appraisals, fear, harm appraisals.

Differences between image type conditions were modeled with two dummy code variables where neutral images served as the reference group. Specifically, this yielded a dummy variable testing the mean difference in ratings (e.g., disgust) between neutral and infection images, and another dummy variable testing the mean difference between neutral and harm images. The dummy variables were modeled as fixed effects. To test the difference between infection and harm images, the same exact models were tested again but with the dummy coding being changed so that infection images served as the reference group. Overall, this approach yielded eight multilevel models overall, with two models per rating measure. The same control variables that were used in the focal mediation analyses (see Methods in main text) were entered as covariates into each multilevel model, to account for the effects of demographic factors and image set on the ratings. Participant was modeled as random intercept to account for the clustering of repeated trials within person.

Models were fit using restricted maximum likelihood, and fixed effects were statistically tested with parametric bootstrapping (10,000 iterations) using the lme4 package (Bates et al., 2014) in RStudio. Bootstrapping was used because it relaxes the assumptions about the residuals (i.e., differences between observed and fitted scores). This approach instead resamples from a Gaussian distribution of parameters generated from the initial model to obtain two-tailed 95% CIs. The approach also provides less biased tests despite the non-normality of some ratings score distributions.

Supplemental Table 2 contains the fixed effects coefficients, i.e., standardized betas (β), that tested the rating differences between image type conditions. These effects suggest that the image task was effective an activating the behavioral immune and harm avoidance systems since all negative emotional ratings were significantly higher for the threat images relative to the neutral images. Specifically, disgust, sickness appraisals, fear, and harm appraisals were each rated significantly higher for the infection and bodily harm images than they were for the neutral images. The next set of results speaks to the specificity of the behavioral immune system to infection stimuli relative to immediate bodily threats, which more strongly activated the harm avoidance system. Specifically, we found that (i) infection images produced higher disgust and sickness appraisals (common markers of behavioral immunity) than did the bodily harm images, and (ii) bodily harm images produced higher fear and harm appraisals (common markers of harm avoidance) than did the infection images.

Supplemental Table 1. Descriptive statistics for rating measures by image type.

|  |  |  | **Raw (non-transformed) units** | | | | |  | **Box-Cox transformed units** | | | | |
| --- | --- | --- | --- | --- | --- | --- | --- | --- | --- | --- | --- | --- | --- |
| **Rating Measure** | **Image Type** | **N** | **Mean** | **SD** | **Min** | **Median** | **Max** |  | **Mean** | **SD** | **Min** | **Median** | **Max** |
| **Disgust** | Neutral | 734 | 14.16 | 20.61 | 0 | 4.28 | 95.23 |  | 1.7 | 1.87 | 0 | 0.84 | 6.42 |
|  | Infection | 734 | 54.4 | 21.26 | 0 | 57 | 98.17 |  | 4.71 | 1.26 | 0 | 5.05 | 6.47 |
|  | Harm | 734 | 36.57 | 24.48 | 0 | 35.13 | 94.93 |  | 3.51 | 1.83 | 0 | 3.72 | 6.41 |
| **Sickness Appraisal** | Neutral | 734 | 18.32 | 20.03 | 0 | 10.67 | 94.83 |  | 2.5 | 2.1 | 0 | 1.77 | 7.9 |
|  | Infection | 734 | 58.18 | 18.94 | 2.7 | 60.95 | 98.67 |  | 5.96 | 1.31 | 0.49 | 6.31 | 8 |
|  | Harm | 734 | 35.05 | 24.67 | 0 | 29.82 | 100 |  | 4.08 | 2.28 | 0 | 4.16 | 8.05 |
| **Fear** | Neutral | 734 | 14.65 | 21.03 | 0 | 4.55 | 94.4 |  | 1.5 | 1.59 | 0 | 0.8 | 5.25 |
|  | Infection | 734 | 31.36 | 25.88 | 0 | 26.88 | 96.93 |  | 2.65 | 1.67 | 0 | 2.72 | 5.28 |
|  | Harm | 734 | 48.76 | 26.36 | 0 | 54.37 | 98.23 |  | 3.62 | 1.41 | 0 | 4.15 | 5.3 |
| **Harm Appraisal** | Neutral | 734 | 18.65 | 20.03 | 0 | 11.4 | 96.37 |  | 2.78 | 2.31 | 0 | 2.04 | 8.86 |
|  | Infection | 734 | 37.38 | 24.37 | 0 | 36.08 | 96.77 |  | 4.75 | 2.42 | 0 | 5.04 | 8.88 |
|  | Harm | 734 | 67.62 | 17.61 | 1 | 70.33 | 100 |  | 7.14 | 1.26 | 0.56 | 7.42 | 8.99 |

Supplementary Table 2. Fixed effects testing mean differences in emotion ratings between image types

|  | **β [95%CI]** | | |
| --- | --- | --- | --- |
|  | **Neutral vs. Infection** | **Neutral vs. Harm** | **Infection vs. Harm** |
| ***Disgust*** | 0.553 [0.547, 0.559]* | 0.332 [0.326, 0.338]* | -0.221 [-0.227, -0.215]* |
| ***Sickness Appraisals*** | 0.529 [0.523, 0.536]* | 0.242 [0.235, 0.248]* | -0.287 [-0.294, -0.281]* |
| ***Fear*** | 0.255 [0.249, 0.260]* | 0.472 [0.467, 0.478]* | 0.218 [0.212, 0.223]* |
| ***Harm Appraisals*** | 0.269 [0.262, 0.275]* | 0.592 [0.586, 0.599]* | 0.323 [0.317, 0.330]* |

Two-tailed 95% CIs are in brackets, determined based on parametric bootstrapping (10,000 iterations). The fixed effect is statistically significant if the CI excludes zero.

* two-tailed *p* < 0.05

Supplementary Table 3. Bootstrapped differences in paths and indirect effects between image types

|  | **Disgust** |  |  | **Sickness Appraisals** |  |  |
| --- | --- | --- | --- | --- | --- | --- |
|  | **N - I** | **N - H** | **I - H** | **N - I** | **N - H** | **I - H** |
| ***a paths***: Diagnosis 🡪 Disgust/Sickness Appraisal | 0.14 [0.06, 0.21]* | 0.06 [0.008, 0.11]* | -0.08 [-0.13, -0.02]* | 0.08 [-0.0002, 0.16] | 0.03 [-0.03, 0.09] | -0.05 [-0.12, 0.01] |
| ***b paths***: Disgust/Sickness Appraisal 🡪 Pandemic Disruption | -0.10 [-0.17, -0.02]* | -0.06 [-0.12, 0.005] | 0.04 [-0.02, 0.10] | -0.06 [-0.13, 0.01] | -0.02 [-0.08, 0.04] | 0.04 [-0.04, 0.11] |
| **a*b indirect effects** | 0.03 [0.001, 0.06]* | 0.01 [-0.01, 0.03] | -0.02 [-0.04, -0.002]* | 0.01 [-0.01, 0.03] | 0.003 [-0.01, 0.02] | -0.01 [-0.03, 0.01] |

N= Neutral, I = Infection, H= Harm.

Two-tailed 95% CIs are in brackets, determined based on wild bootstrapping (10,000 iterations). The difference is statistically significant if the CI excludes zero.

* two-tailed *p* < 0.05

**Harm image effects without snakes and spiders**

Path coefficients and mediational effects were re-estimated for harm images after removing all images depicting snakes and spiders. These results are depicted in Supplemental Figures 1 and 2, with the updated harm image effects highlighted.


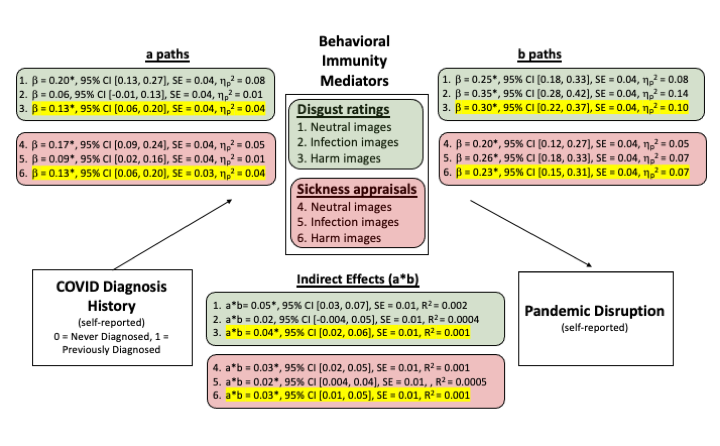


Supplemental Figure 1. Behavioral immunity effects without snake and spider images.


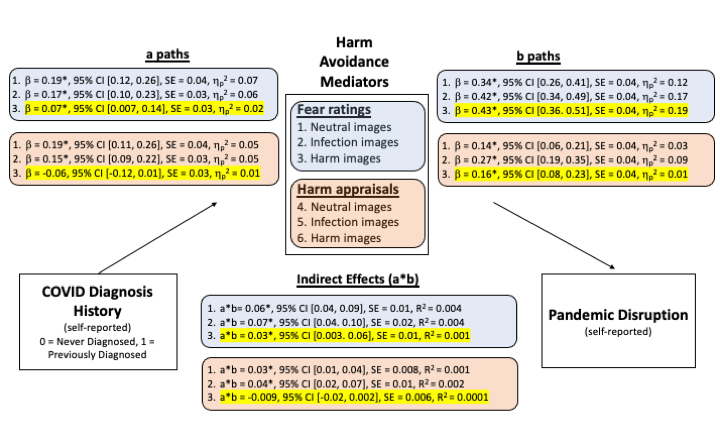


Supplemental Figure 2. Harm avoidance effects without snake and spider images.
